# Comparative Effects of Post-Extrusion Annealing and PLLA Surface Modification on the Degradation Behavior and Endothelial Responses of ZK60 Magnesium Alloy

**DOI:** 10.1021/acsomega.6c02648

**Published:** 2026-06-02

**Authors:** Guan-Lin Wu, Chin-En Yen, Ming-Long Yeh

**Affiliations:** † Department of Biomedical Engineering, 34912 National Cheng Kung University, No. 1, Daxue Rd., East Dist., Tainan City 701401, Taiwan; ‡ Medical Device Innovation Center, National Cheng Kung University, No. 1, Daxue Rd., East Dist., Tainan City 701401, Taiwan

## Abstract

Biodegradable magnesium alloys have emerged as attractive materials for cardiovascular stents due to their mechanical compatibility with vascular tissues and inherent biocompatibility. Despite these advantages, their clinical translation is hindered by rapid and localized degradation in physiological environments. To address this challenge, the present study systematically compares the effects of bulk postprocessing and surface modification on the degradation behavior and biological performance of extruded ZK60 magnesium alloy. Hot-extruded ZK60 (E-ZK), postextrusion annealed ZK60 (A-ZK), and poly­(l-lactic acid)-coated ZK60 (P-ZK) were investigated through a stepwise experimental design. The influence of annealing on intrinsic mechanical properties was first examined, followed by electrochemical analysis, immersion degradation testing, and endothelial cell evaluations across all material conditions. While postextrusion annealing altered the mechanical response of ZK60, it showed limited effectiveness in mitigating degradation or enhancing endothelial interactions. In contrast, the PLLA surface coating significantly reduced electrochemical reactivity. It decreased the corrosion current density from approximately 151–223 μA/cm^2^ to around 22 μA/cm^2^. Additionally, the corrosion rate was reduced from approximately 3.46–5.09 mm/year to about 0.51 mm/year. This enhanced corrosion resistance contributed to improved surface integrity and more favorable endothelial responses, with cell viability reaching approximately 140% on day 3 for P-ZK, compared to around 125% for the uncoated samples. Collectively, these results demonstrate that surface modification plays a more decisive role than bulk annealing in optimizing degradation control and endothelial compatibility of magnesium-based biodegradable stents.

## Graphical abstract

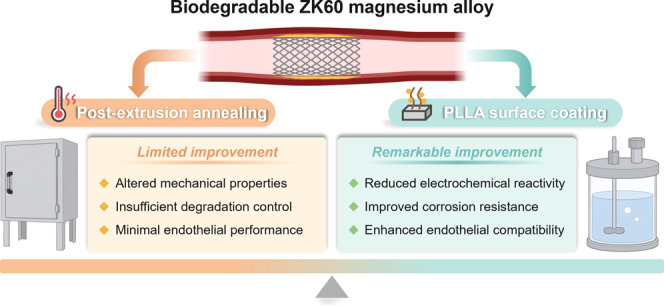

## 1 Introduction

Coronary heart disease (CHD) continues to impose a substantial global health burden, arising primarily from the progressive accumulation of atherosclerotic plaques that restrict coronary perfusion and precipitate myocardial ischemia.^1–3^ Percutaneous coronary intervention (PCI) has thus become a primary clinical strategy for re-establishing vascular patency in affected patients.^4–6^ While the introduction of vascular stents has significantly improved procedural success and short-term outcomes, the permanent implantation of metallic stents is accompanied by well-recognized long-term complications, including in-stent restenosis, impaired endothelial recovery, and late stent thrombosis.^7,8^ Although drug-eluting stents have mitigated restenosis by suppressing neointimal hyperplasia, delayed re-endothelialization and the need for prolonged dual antiplatelet therapy remain unresolved clinical concerns.^9,10^

Bioresorbable metallic stents have therefore emerged as an alternative paradigm, aiming to provide temporary mechanical scaffolding during vascular healing while ultimately eliminating long-term implant-related risks through controlled degradation.^11–13^ Among available biodegradable metals, magnesium-based alloys have attracted particular attention due to their favorable biocompatibility, high specific strength, and inherent biodegradability.^14–16^ However, the high chemical reactivity of magnesium in physiological environments presents a critical limitation. Accelerated degradation can lead to premature loss of mechanical support and the formation of localized alkaline and hydrogen-rich microenvironments, potentially impairing endothelial function and vascular healing.^17–19^

ZK60 magnesium alloy, containing zinc and zirconium as alloying elements, has been extensively investigated for biomedical use owing to its refined microstructure and relatively high mechanical strength compared with many other magnesium alloys.^20,21^ Hot extrusion is commonly employed to further enhance its mechanical performance via grain refinement, while postextrusion annealing is frequently used to relieve residual stresses and adjust microstructural characteristics.^22^ Previous studies have shown that annealing can significantly affect grain size, residual stress levels, and the distribution of secondary phases in magnesium alloys, which in turn impacts both mechanical performance and corrosion behavior. However, the effectiveness of annealing in improving resistance to degradation can vary widely and depend heavily on specific processing conditions.^23–25^ While thermomechanical treatments are known to affect the intrinsic mechanical properties of materials, their effectiveness in regulating degradation behavior is also important. Specifically, it is crucial to investigate the degradation behavior of ZK60 magnesium alloy and its implications for mechanical stability and endothelial responses under physiological conditions.^26,27^ In particular, whether postextrusion annealing alone can meaningfully suppress localized corrosion processes that govern both structural integrity and vascular cell responses has not been clearly established.

Beyond bulk material processing, surface modification has emerged as a complementary strategy to address the degradation challenges of magnesium alloys.^28,29^ By directly tailoring the material–environment interface, surface coatings offer the potential to modulate electrochemical reactions and local chemical conditions without substantially altering the bulk mechanical properties of the substrate.^30,31^ Among the various strategies explored, biodegradable polymer coatings, especially poly­(l-lactic acid) (PLLA), have demonstrated significant potential in reducing corrosion rates and improving the cytocompatibility of magnesium-based materials.^32–34^ Previous research has shown that PLLA coatings can effectively lower the corrosion rate of magnesium alloys by acting as a physical barrier and modulating the interface between the material and its environment.^35,36^ Additionally, PLLA coatings have been found to enhance cytocompatibility by stabilizing local pH levels and reducing rapid ion release.^37,38^ However, most existing studies have primarily focused on surface modification techniques in isolation, without systematically assessing their effectiveness compared to bulk microstructural modification methods. As a result, the extent to which surface modifications can either compensate for or interact synergistically with the intrinsic bulk properties of the material remains inadequately understood.

Accordingly, the present study aims to clarify the relative roles of bulk postextrusion annealing and PLLA surface modification in governing the degradation behavior and endothelial responses of ZK60 magnesium alloy. These two modification strategies were examined separately to clarify their individual contributions to degradation behavior and biological response. The effects of postextrusion annealing were first examined by comparing extruded ZK60 (E-ZK) and annealed ZK60 (A-ZK) with respect to intrinsic mechanical properties and degradation behavior under simulated physiological conditions. Subsequently, PLLA-coated ZK60 (P-ZK) was prepared to evaluate how surface modification influences electrochemical behavior, immersion degradation, and in vitro endothelial cell responses. Through this stepwise comparative framework, this work seeks to provide mechanistic insight into how bulk processing and interface-focused surface engineering jointly contribute to degradation control and vascular biocompatibility in magnesium-based biodegradable stent materials.

## 2 Materials and Methods

### 2.1 Material Preparation and Heat Treatment

ZK60 magnesium alloy billets were fabricated by melting high-purity magnesium, with controlled additions of 6.0 wt % zinc and 0.6 wt % zirconium, followed by casting into cylindrical billets. The billets were subsequently hot-extruded into rods with a final diameter of 12 mm (AMSpec, Taiwan). These hot-extruded samples are denoted as extruded ZK60 (E-ZK).

To examine the influence of postextrusion annealing on the mechanical and degradation characteristics of the alloy, a subset of the extruded rods was subjected to a secondary heat treatment. The rods were heated to 400 °C at a rate of 5 °C/min, maintained at this temperature for 8 h, and then furnace-cooled to ambient temperature. The selected annealing parameters were based on the Mg–Zn binary phase diagram, within the range between the single-phase and dual-phase regions, where microstructural homogenization and phase redistribution are expected to occur.^39^ Samples processed by this annealing protocol are referred to as annealed ZK60 (A-ZK).

### 2.2 Polymer Coating Application

Poly­(l-lactic acid) (PLLA; Sigma-Aldrich, America) was dissolved in dichloromethane to prepare a 5 wt % solution under magnetic stirring at room temperature until complete dissolution.

The PLLA coating was applied onto mechanically polished E-ZK substrates using a microdrop coating method. Approximately 200 μL of the PLLA solution was dispensed onto each specimen to ensure complete surface coverage. The solution was allowed to spread naturally over the surface to form a uniform coating layer.

After the coating process, the samples were placed in a vacuum chamber and dried for 12 h at room temperature to ensure complete solvent evaporation and stabilization of the polymer layer. These coated samples are designated as the P-ZK group.

### 2.3 Surface Characterization

Specimens from the E-ZK and A-ZK groups were sectioned into discs with a thickness of 5 mm. The samples were mechanically ground using silicon carbide abrasive papers with grit sizes ranging from #120 to #2500. P-ZK specimens were prepared by applying the PLLA coating onto ground E-ZK substrates. All samples were ultrasonically cleaned in ethanol, rinsed with deionized water, and air-dried prior to characterization.

Surface morphology was examined using field-emission scanning electron microscopy (FE-SEM; SU5000, Hitachi, Japan). Elemental composition was analyzed by energy-dispersive X-ray spectroscopy (EDS; EDAX, USA).

### 2.4 Mechanical Testing

#### 2.4.1 Hardness Test

Hardness measurements of the E-ZK and A-ZK samples were conducted using a Vickers hardness tester (HMV-G20S, Shimadzu, Japan) under a 1 kg load applied for 10 s. The Vickers hardness (HV) was calculated according to the standard formula based on the mean diagonal length of the indentations (eq 1), where *F* represents the applied load (N), *S* denotes the indentation surface area (mm^2^), *g* is the standard gravitational acceleration, and *d* corresponds to the average diagonal length of the indentation (mm).

1 $$HV=\frac{F}{Sg}=\frac{2F\;\sin\left(\frac{136^{{}^{\circ}}}{2}\right)}{gd^{2}}\approx 0.1891\frac{F}{d^{2}}\left[N/mm^{2}\right]$$

#### 2.4.2 Tensile Test

Uniaxial tensile tests were conducted in accordance with ASTM standards using a hydraulic universal testing machine equipped with an extensometer (MTS 810, USA). Dog-bone-shaped specimens were prepared following standard geometries. Testing was performed at a constant crosshead speed of 0.1 mm/s, and load–displacement data were continuously recorded. Engineering stress–strain curves were constructed to determine yield strength, ultimate tensile strength, and elongation to failure.

### 2.5 Corrosion Evaluation

#### 2.5.1 Electrochemical Test

Electrochemical measurements were conducted using a conventional three-electrode system, with the specimen serving as the working electrode, a saturated calomel electrode as the reference electrode, and a platinum electrode as the counter electrode (Vertex.One.EIS, Ivium, Holland). All electrodes were immersed in simulated body fluid (SBF) and allowed to stabilize until the open-circuit potential reached equilibrium. Potentiodynamic polarization curves were recorded at a scan rate of 1 mV/s, and the corrosion potential (*E*_corr_) and corrosion current density (*I*_corr_) were determined using Tafel extrapolation. The corrosion rate (CR) was calculated according to (eq 2), where EW (g) is the equivalent weight and ρ (g/cm^3^) is the density.

2 $$CR\left(mm/year\right)=3.27\times 10^{3}\times \frac{EW}{\rho }\times I_{corr}$$

The SBF solution used in this study was prepared by dissolving reagent-grade chemicals in deionized water to achieve ion concentrations similar to human plasma. The following chemicals were used: sodium chloride (5.40 g/L), sodium bicarbonate (0.73 g/L), sodium carbonate (2.03 g/L), potassium chloride (0.23 g/L), potassium hydrogen phosphate (0.18 g/L), magnesium chloride (0.31 g/L), HEPES (11.9 g/L), calcium chloride (0.29 g/L), and sodium sulfate (0.07 g/L). Once all the chemicals were dissolved, the pH of the solution was adjusted to 7.4 using 1 M sodium hydroxide.

#### 2.5.2 Hydrogen Evolution Test

The hydrogen evolution test was conducted using a drainage gas collection system, the specimen was immersed in SBF for 9 h, and the scale drop of the buret was recorded at regular intervals to measure the amount of hydrogen gas released. This short-term test was designed to evaluate the initial corrosion kinetics and early stage degradation behavior immediately after exposure.

#### 2.5.3 Immersion Test

According to ASTM G31-21, the specimen surface area to solution volume ratio was set to 20 mL/cm^2^. The specimens were immersed in SBF and placed in a circulating constant-temperature oven at 37 °C. To evaluate the longer-term degradation behavior, immersion tests were conducted for 1, 3, and 5 days. After immersing the samples for the specified durations, they were removed from the SBF, gently rinsed with deionized water, and dried for surface analysis. The purpose of the immersion test in this study was to examine morphological changes and the deposition of biologically relevant elements on the surfaces of the samples.

### 2.6 In Vitro Test

#### 2.6.1 Cell Culture

In the in vitro tests, the human endothelial cell line (EA.hy926; CRL-2922, ATCC, America) was utilized to evaluate the reaction to the materials. The cells were cultured in Dulbecco’s Modified Eagle’s Medium (DMEM; 12430054, Thermo Fisher Scientific, America) supplemented with 10% fetal bovine serum under controlled conditions of 5% CO_2_ at 37 °C.

#### 2.6.2 Cell Viability

The material extract was prepared according to ISO 10993-5, filtered, and supplemented with fetal bovine serum for cell viability testing. The experimental group was treated with the material extract, the negative control (NC) group was treated with the original cell culture medium, and the positive control group was treated with culture medium containing dimethyl sulfoxide.

Cell viability was assessed after 1, 3, and 5 days of incubation, quantifying using the Cell Counting Kit-8 assay (CCK8; Sigma-Aldrich, America), and absorbance at 450 nm was measured using an ELISA reader (EP-800, Bio-Tek, America). Viability was standardized to the NC group at each time point, with the control value for each day set to 100%. The relative cell viability was calculated using the following eq (eq 3)­

3 $$cell\;viability\left(\%\right)=\frac{{OD}_{test}-{OD}_{blank}}{{OD}_{NC}-{OD}_{blank}}\times 100\%$$

#### 2.6.3 Cell Adhesion

Cells were seeded onto the surface of the specimens and placed in an incubator for 3 and 24 h for adhesion. After the incubation period, the specimens were fixed in 4% paraformaldehyde for 1 h, followed by washing with phosphate-buffered saline (PBS). The specimens were then dehydrated using a gradient ethanol series and hexamethyldisilazane, and air-dried. The cell adhesion morphology was observed using SEM.

#### 2.6.4 Cell Migration

The Culture-Insert 2 Well cell culture dish (ibidi, Germany) was used for the experiment. After seeding the cells and incubating for 24 h, the inset well was removed to create a 500 μm cell gap. Material extract was then added for incubation, and cell images were captured every day. A narrower cell gap observed during the experiment indicated better cell migration ability, which would contribute to tissue repair.

### 2.7 Statistical Analysis

Statistical analyses were performed using GraphPad PRISM 9 software. The quantitative data are expressed as the mean. For comparisons between two groups, an independent Student’s *t*-test was employed. For comparisons involving more than two groups, a one-way analysis of variance followed by Tukey’s posthoc test was utilized. A *P*-value less than 0.05 was regarded as statistically significant.

## 3 Results

### 3.1 Surface Morphology and Element Composition

E-ZK and A-ZK exhibited comparably smooth, planar surfaces with polishing marks remaining from mechanical preparation (Figure 1A,B). In contrast, P-ZK showed complete masking of polishing traces by a continuous PLLA coating layer (Figure 1C). The coating contained abundant micron-scale pores, consistent with solvent (dichloromethane) evaporation and associated void formation during drying.

**Figure 1:**
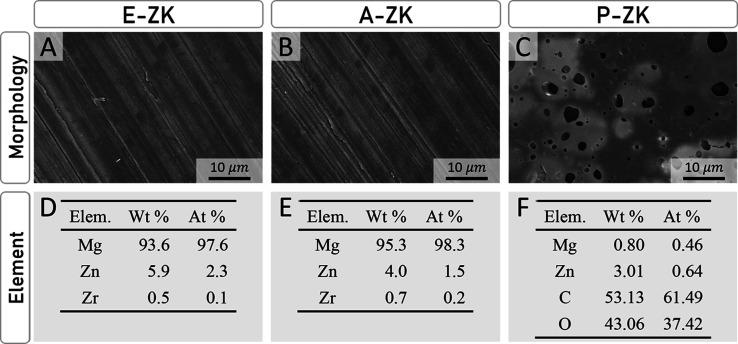
Surface morphology and elemental composition of ZK60 samples. (A) Smooth and flat surface morphology of E-ZK. (B) Comparable smooth surface observed on A-ZK. (C) P-ZK showing a porous coating layer covering the substrate surface. (D) E-ZK confirming the characteristic elemental composition of ZK60 alloy. (E) A-ZK showing a similar elemental distribution to E-ZK. (F) P-ZK characterized by increased carbon and oxygen signals and reduced magnesium content, indicating effective PLLA surface coverage.

EDS analysis confirmed that E-ZK and A-ZK retained the characteristic ZK60 composition, with ∼6 wt % zinc and ∼1 wt % Zr (Figure 1D,E). For P-ZK, magnesium and zinc signals were markedly reduced, while carbon and oxygen increased substantially (Figure 1F), indicating effective PLLA coverage of the alloy surface. After applying the PLLA coating, the reduced ratio of magnesium signals to zinc signals can be attributed to the more uniform distribution of magnesium within the matrix, which is better masked by the polymer coating. In contrast, zinc is mainly linked to localized secondary phases, which may still be partially detectable through thinner areas of the coating or surface pores.

### 3.2 Mechanical Properties

The mechanical properties were measured prior to immersion to reflect the intrinsic material response after processing (Table 1). Vickers hardness testing indicated higher hardness for E-ZK than A-ZK. The indentation diagonal was ∼170 μm for E-ZK and ∼185 μm for A-ZK under identical loading, corresponding to hardness values of ∼63 HV and ∼53 HV, respectively.

**Table 1:** Mechanical Properties of E-ZK and A-ZK^a^

	Vickers hardness (HV)	Young’s modulus (GPa)	Yield strength (MPa)	Tensile strength (MPa)	Elongation at break (%)
E-ZK	63.10	46.7	115.28	252.94	9.45
A-ZK	53.23	44.1	98.41	234.84	9.74

a The intrinsic mechanical properties of E-ZK and A-ZK measured immediately after processing, reflecting the influence of post-extrusion annealing on the mechanical response of ZK60 prior to immersion.

Engineering stress–strain curves for E-ZK and A-ZK showed broadly similar profiles. The Young’s modulus was slightly higher for E-ZK (46.7 GPa) than A-ZK (44.1 GPa). Using the 0.2% offset method, yield strength decreased from ∼115 MPa (E-ZK) to ∼98 MPa (A-ZK). Ultimate tensile strength likewise decreased from ∼253 MPa (E-ZK) to ∼235 MPa (A-ZK). In contrast, elongation at break increased slightly following annealing (9.45% for E-ZK vs 9.74% for A-ZK), consistent with a modest ductility improvement.

### 3.3 Corrosion Behavior

#### 3.3.1 Potentiodynamic Polarization

Polarization curves showed overlapping behavior for E-ZK and A-ZK, whereas P-ZK exhibited a clear shift toward improved corrosion resistance (Figure 2). Consistent with this trend, *E*_corr_ was −1.33 V for E-ZK and −1.35 V for A-ZK, while P-ZK increased to −0.48 V (Table 2). Likewise, *I*_corr_ increased from ∼151 μA/cm^2^ (E-ZK) to ∼223 μA/cm^2^ (A-ZK) but decreased substantially to ∼22 μA/cm^2^ for P-ZK. The calculated corrosion rate was ∼3.46 mm/year (E-ZK) and ∼5.09 mm/year (A-ZK), compared with ∼0.51 mm/year for P-ZK.

**Figure 2:**
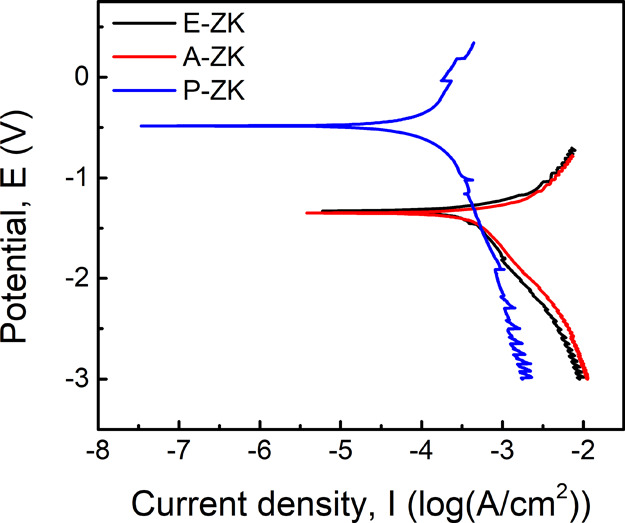
Electrochemical corrosion behavior of ZK60 samples. Potentiodynamic polarization curves of E-ZK, A-ZK, and P-ZK, illustrating comparable corrosion behavior between E-ZK and A-ZK, while P-ZK exhibited a reduced corrosion current density and a shifted corrosion potential.

**Table 2:** Electrochemical Parameters of ZK60 Samples^a^

	potential (V)	current density (μA/cm2)	resistance (Ω)	corrosion rate (mm/year)
E-ZK	–1.33	151.10	165.40	3.46
A-ZK	–1.35	222.50	112.30	5.09
P-ZK	–0.48	22.09	1132.00	0.51

a Parameters obtained from potentiodynamic polarization curves of E-ZK, A-ZK, and P-ZK used to evaluate the effect of post-extrusion annealing and PLLA surface modification on corrosion behavior.

#### 3.3.2 Hydrogen Evolution

Hydrogen evolution profiles distinguished the three conditions (Figure 3). E-ZK displayed a relatively steady increase of approximately 0.1 mL/cm^2^ per hour. A-ZK exhibited a strong time-dependent acceleration, reaching ∼3.0 mL/cm^2^ per hour at 9 h. In contrast, P-ZK showed substantially lower hydrogen evolution with a gradual trend throughout immersion.

**Figure 3:**
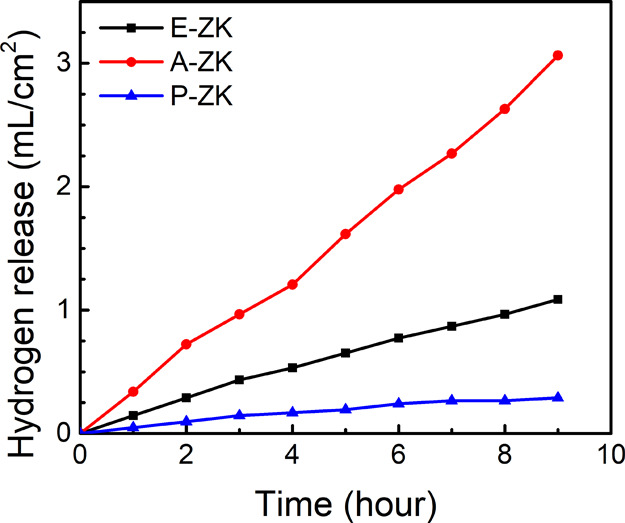
Hydrogen evolution of ZK60 samples during immersion. Time-dependent hydrogen evolution profiles of E-ZK, A-ZK, and P-ZK, showing a markedly lower hydrogen evolution rate for P-ZK compared with uncoated samples.

#### 3.3.3 Immersion Degradation and Surface Chemistry

After 1 day, E-ZK surfaces showed widespread cracking (Figure 4A), while A-ZK presented finer cracks with visible white precipitates (Figure 4B). P-ZK largely retained its preimmersion appearance (Figure 4C). EDS revealed newly prominent oxygen, phosphorus, and calcium on both E-ZK and A-ZK (Figure 4D,E), whereas P-ZK exhibited markedly lower phosphorus and calcium (Figure 4F).

**Figure 4:**
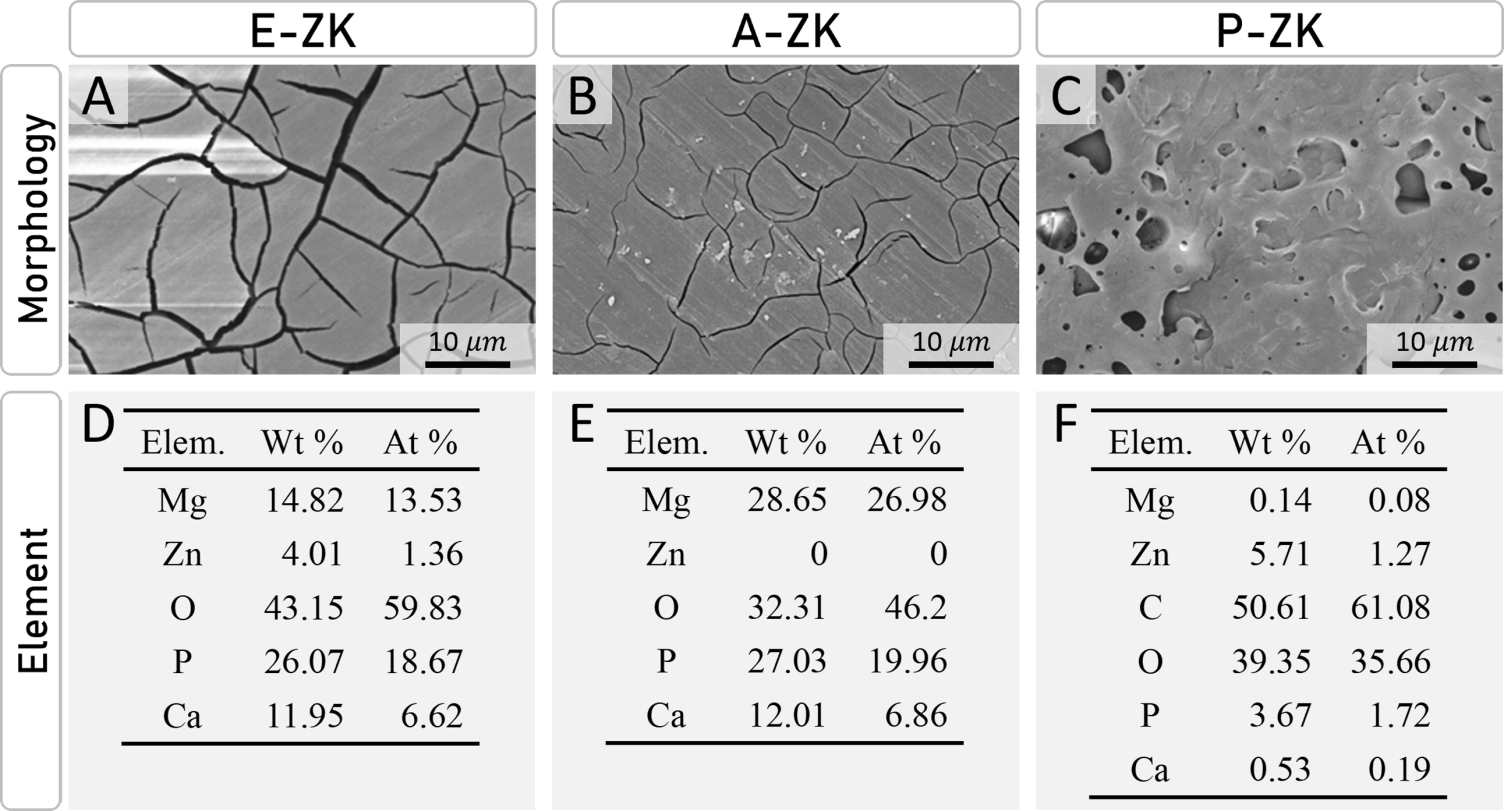
Surface morphology and elemental composition after 1 day immersion. (A) Surface cracking observed on E-ZK after 1 day of immersion. (B) Finer surface cracks and precipitate formation on A-ZK. (C) P-ZK maintaining a surface morphology similar to the preimmersion state. (D) E-ZK showing the emergence of oxygen, phosphorus, and calcium. (E) A-ZK showing the presence of degradation-related elements on the surface. (F) P-ZK revealing minimal deposition of degradation-related elements.

Additionally, in the P-ZK group, the relatively high zinc signal observed after 1 day of immersion is likely due to localized surface effects rather than a general increase in degradation. The limited penetration of the electrolyte through the PLLA coating may expose zinc-containing secondary phases in certain areas, leading to localized increases in zinc signals in the EDS measurements.

After 3 days, E-ZK showed increased crack density with propagation into grain interiors (Figure 5A). A-ZK retained fine microcracks with limited change in distribution (Figure 5B). P-ZK showed slight enlargement of coating pores (Figure 5C). EDS analysis of E-ZK revealed the presence of magnesium-, phosphorus-, and calcium-containing corrosion products (Figure 5D). A-ZK retained comparatively higher magnesium signals (Figure 5E). P-ZK continued to show low phosphorus and calcium signals (Figure 5F).

**Figure 5:**
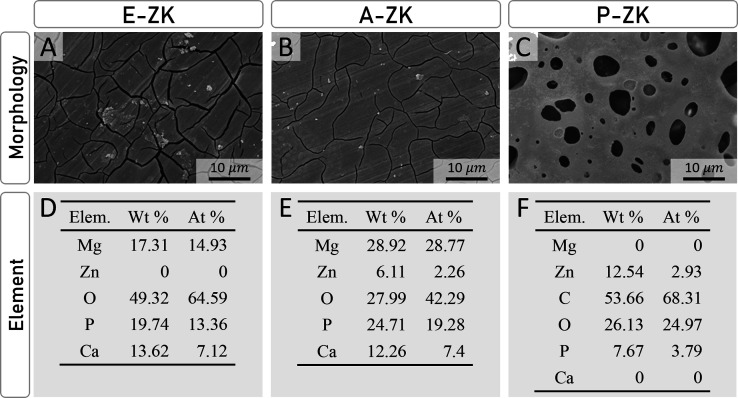
Surface morphology and elemental composition after 3 day immersion. (A) Increased crack density and propagation across grain interiors on E-ZK. (B) Persistent fine microcracks on A-ZK with limited morphological change. (C) Slight enlargement of surface pores on P-ZK. (D) E-ZK indicating the presence of magnesium-, phosphorus-, and calcium-containing corrosion products. (E) A-ZK showing relatively higher surface magnesium content compared with E-ZK. (F) P-ZK confirming low phosphorus and calcium deposition.

After 5 days, E-ZK exhibited extensive crack widening and interconnection, obscuring the original grain structure; large degradation products accumulated along crack regions (Figure 6A). A-ZK showed deeper, wider cracks with pronounced product accumulation (Figure 6B). P-ZK maintained a continuous porous coating morphology, and the coating appeared denser than prior to immersion (Figure 6C). This change may be linked to structural alterations in the polymer during exposure to the solution. EDS analysis revealed the presence of magnesium-, phosphorus-, and calcium-containing corrosion products on E-ZK (Figure 6D). A-ZK showed elevated zinc signals (Figure 6E). P-ZK exhibited only limited magnesium- and phosphorus-containing species and no detectable calcium (Figure 6F).

**Figure 6:**
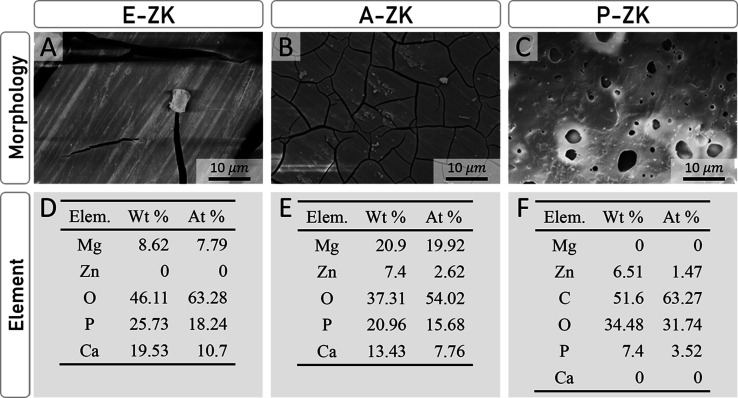
Surface morphology and elemental composition after 5 day immersion. (A) Extensive crack widening and loss of discernible grain structure on E-ZK. (B) Deepened and widened cracks with substantial degradation product accumulation on A-ZK. (C) P-ZK maintaining a continuous and porous coating morphology after prolonged immersion. (D) E-ZK degradation products composed of magnesium-, phosphorus-, and calcium-containing oxides. (E) A-ZK detected elevated zinc content, indicating degradation dominated by secondary phases. (F) P-ZK showing limited magnesium- and phosphorus-containing species and absence of calcium.

### 3.4 Cell Compatibility

#### 3.4.1 Cell Viability

Cell viability was normalized to the negative control (NC, 100%) (Figure 7). At day 1, E-ZK and A-ZK were ∼94%, while P-ZK was ∼100%; no significant differences relative to NC were observed. At day 3, viability increased to ∼125% for both E-ZK and A-ZK, and to ∼140% for P-ZK; all material groups were significantly higher than NC, with P-ZK also significantly higher than the uncoated groups. By day 5, all three material groups converged to ∼140% and remained significantly above NC, with no significant differences among materials. It is important to emphasize that cell viability values surpassing 100% reflect an enhancement in metabolic activity relative to the NC group, rather than an increase in cell quantity, given that the assay quantifies metabolic function.

**Figure 7:**
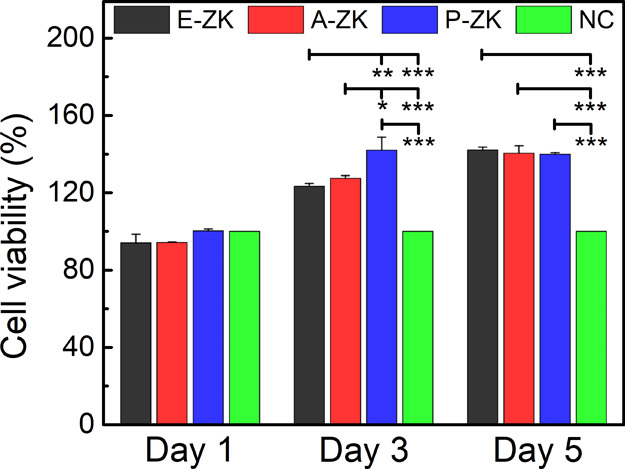
Endothelial cell viability of ZK60 samples. The relative viability of endothelial cells cultured with extracts from E-ZK, A-ZK, and P-ZK was assessed after 1, 3, and 5 days. The results were normalized to the NC group for each corresponding time point, with the control for each day defined as 100%. (Statistical significance between groups is indicated by asterisks with *P < 0.05, **P < 0.01, ***P < 0.001).

#### 3.4.2 Cell Adhesion

At 3 h, endothelial cells adhered to E-ZK with predominantly rounded morphology and visible lamellipodia (Figure 8A). A-ZK showed a comparable pattern (Figure 8B). P-ZK exhibited a higher number of adhered cells with early intercellular connections evident (Figure 8C). At 24 h, E-ZK and A-ZK surfaces largely lacked intact cell bodies, with mainly residual adhesion traces and debris observed (Figure 8D,E). This phenomenon may relate to the local microenvironment at the material surface during direct contact conditions. In contrast, P-ZK retained adherent cells with clear spreading and extensive lamellipodial extension (Figure 8F).

**Figure 8:**
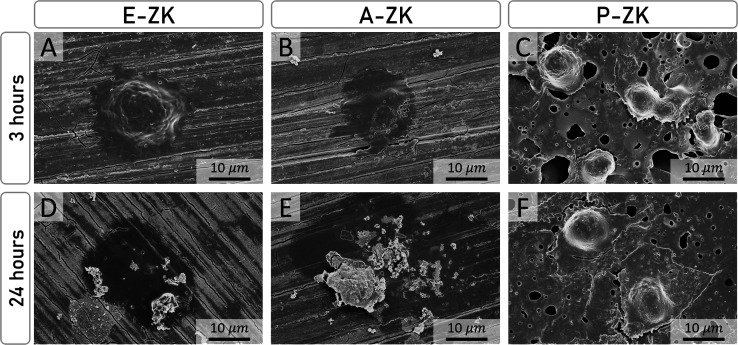
Endothelial cell adhesion on material surfaces. (A–C) Cell adhesion morphology on E-ZK, A-ZK, and P-ZK surfaces after 3 h of incubation. (D–F) Cell adhesion morphology on E-ZK, A-ZK, and P-ZK surfaces after 24 h of incubation, highlighting differences in cell retention and spreading behavior.

#### 3.4.3 Cell Migration

Migration was quantified by monitoring closure of a defined central gap. At 0 h, a clear cell-free gap was present in all groups (Figure 9A–C). After 24 h, inward migration was evident for all conditions, with P-ZK showing more pronounced gap narrowing (Figure 9D–F). After 48 h, E-ZK and A-ZK displayed continued migration, whereas P-ZK exhibited near-complete gap closure (Figure 9G–I).

**Figure 9:**
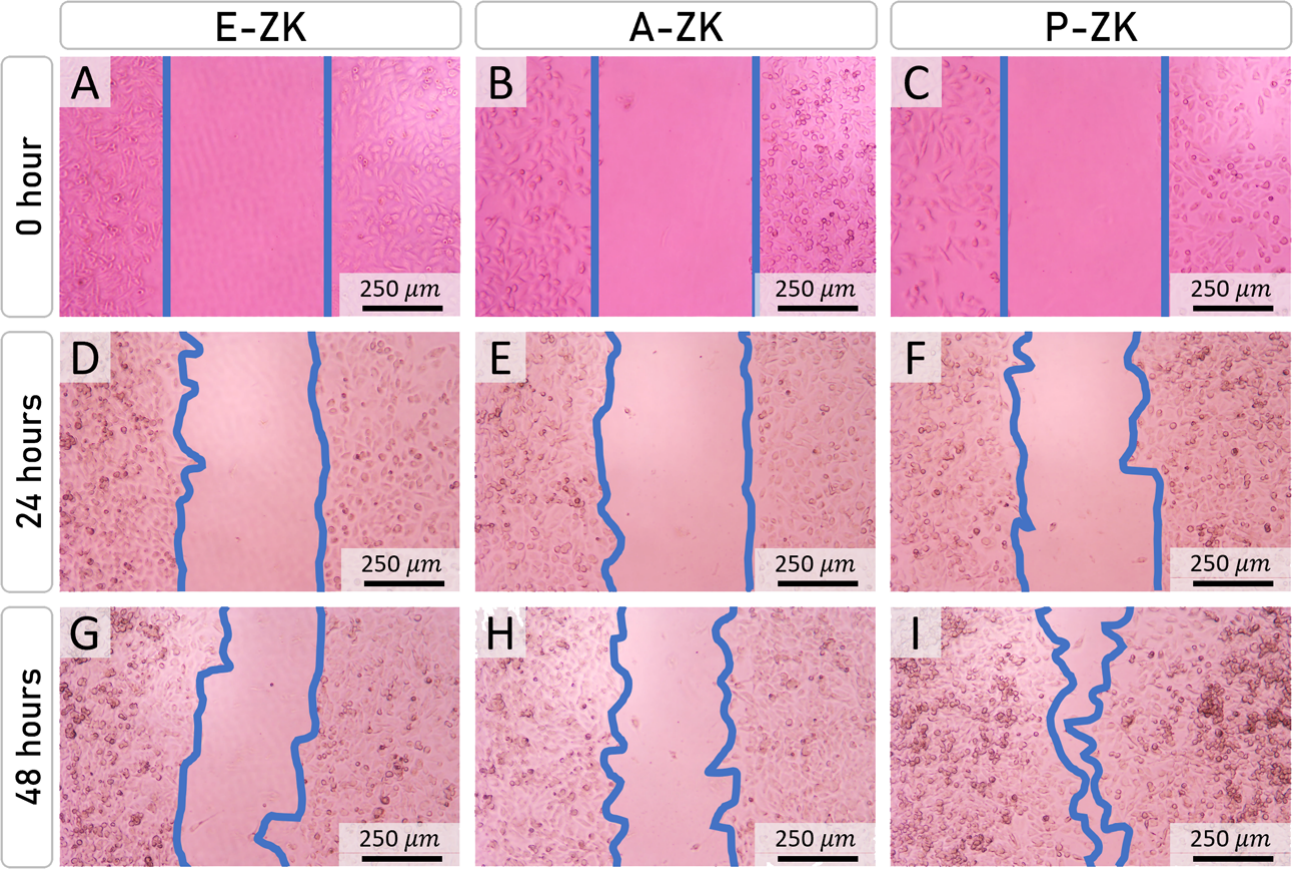
Endothelial cell migration behavior of ZK60 samples. (A–C) Initial cell distribution showing a defined central gap at 0 h. (D–F) Cell migration toward the central gap after 24 h of incubation. (G–I) Migration results after 48 h, showing near-complete gap closure on P-ZK compared with uncoated samples.

## 4 Discussion

### 4.1 Effect of Postextrusion Annealing on Mechanical Response and Degradation Behavior

Postextrusion annealing is widely used in magnesium alloys to relieve residual stress and modify microstructure after severe plastic deformation.^40,41^ The selected annealing parameters fall within the transitional zone between the single-phase and dual-phase regions in the Mg–Zn binary phase diagram.^39^ It should be noted that postextrusion annealing is not expected to fundamentally alter the phase constitution of ZK60 magnesium alloy. Instead, this thermal treatment may lead to the coarsening of secondary phases, which could worsen microgalvanic corrosion and undermine the expected improvements in corrosion resistance.^42,43^ In this study, annealing was applied to extruded ZK60 to evaluate whether such bulk processing could improve mechanical stability and biological performance under physiological conditions.

Conventional mass loss measurements based on ASTM standards require chemical removal of corrosion products, which would inevitably damage the PLLA coating on P-ZK samples and compromise comparability between groups. Therefore, immersion tests were used primarily for qualitative evaluation of surface degradation and elemental deposition, complementing electrochemical measurements.^44^ Prior to immersion, extruded and annealed ZK60 exhibited comparable surface morphology and elemental composition, indicating that annealing did not significantly alter the initial surface elemental composition. During the immersion process, both materials developed surface cracking that progressed with time. Although annealed ZK60 showed slightly reduced cracking at early stages, prolonged exposure led to deeper crack propagation and increased accumulation of degradation products. These findings suggest that annealing was not effective in mitigating localized corrosion under the present conditions. This type of degradation can negatively impact the mechanical integrity of the material. These results are consistent with previous research, which shows that the effects of annealing on magnesium alloys are highly dependent on its influence on the microstructure, particularly grain size and the distribution of secondary phases.^40,45^

Elemental analysis suggests that electrochemically active secondary phases influence the degradation of annealed ZK60. However, zinc signals were detected inconsistently, likely due to the formation of surface corrosion products. These corrosion layers can mask the underlying phases during EDS analysis.^42,44^ The localized detection of zinc at later stages may suggest spatially heterogeneous degradation, where secondary phases are exposed or concentrated in specific regions rather than uniformly across the surface. Consistent with this observation, hydrogen evolution measurements revealed no reduction in electrochemical activity following annealing and even showed increased hydrogen release at prolonged immersion times. Although this study did not conduct direct microstructural characterization, the results imply that annealing did not lead to a sufficiently altered distribution or electrochemical behavior of the relevant phases. Previous research has shown that annealing reduces dislocation density, alleviates internal residual stresses, and promotes grain growth. These changes may weaken the ZK-series alloy’s structural integrity and enhance localized corrosion. Additionally, the potential redistribution or coarsening of secondary phases could intensify microgalvanic interactions, accelerating hydrogen evolution over time.^46–48^

Postextrusion annealing altered the intrinsic mechanical properties of the material by reducing its strength while slightly improving ductility. However, these changes did not appear to enhance resistance to degradation-related surface damage or cracking when exposed to physiological conditions.^49^ Although the degradation behavior was systematically characterized and its implications for mechanical stability were discussed, the study lacked experimental measurements of the actual mechanical performance after exposure to these conditions. Future research will focus on assessing mechanical integrity following degradation to provide a more comprehensive understanding of the structure–function relationship in biodegradable magnesium alloys.

In vitro biocompatibility experiments have revealed a notable discrepancy between poor cell adhesion on E-ZK and A-ZK surfaces and the relatively high cell viability observed in extract-based assays. This discrepancy can be attributed to differences in testing conditions. In direct contact cultures, cells come into direct contact with the material surface. This exposure can lead to rapid degradation of magnesium alloys, resulting in localized changes in the microenvironment, including elevated pH, increased magnesium ion concentrations, and hydrogen gas evolution.^50–52^ These factors can adversely affect cell adhesion, spreading, and morphology. In contrast, extract-based assays assess the averaged effects of degradation products in the culture medium, where ionic concentrations and pH changes are diluted and buffered. As a result, cytotoxicity is generally reduced, leading to higher apparent cell viability. Therefore, the findings from these two types of assays represent different aspects of material–cell interactions and should be interpreted together for a comprehensive understanding.^19,53^

Collectively, these results indicate that postextrusion annealing primarily affects the intrinsic mechanical properties of ZK60 by promoting microstructural recovery and recrystallization. While reducing dislocation density enhances ductility, it may also result in decreased strength and potential grain coarsening. However, under the current annealing conditions, these changes were not sufficient to mitigate localized corrosion or improve endothelial compatibility. Consequently, annealing offers limited advantages in regulating the degradation behavior or biological performance of ZK60 in physiological conditions.

### 4.2 Role of PLLA Coating in Degradation Regulation and Endothelial Response

In contrast to bulk annealing, PLLA surface modification consistently improved both degradation control and endothelial performance of ZK60. The PLLA coating stabilized the material–environment interface throughout immersion, highlighting the effectiveness of surface engineering in mitigating the intrinsic reactivity of magnesium alloys.^54,55^ In this study, PLLA coating was applied to the E-ZK samples instead of the A-ZK samples to isolate the effect of surface modification from bulk microstructural changes. Although the potential synergistic effects of combining annealing with PLLA coating were not investigated in this work, the results provide a clear and systematic comparison between these two commonly used modification strategies.

The electrochemical results indicate a significant reduction in the corrosion rate after applying a PLLA coating. It is important to highlight that the corrosion rates reported for the P-ZK group were measured under conditions where the polymer coating remained largely intact. Previous studies have shown that PLLA coatings can maintain their structural integrity during the initial stages, followed by gradual degradation in physiological environments.^56–58^ Therefore, the notably lower corrosion rate observed in this study primarily reflects the protective effect during the early stage. Preserving mechanical integrity and slowing degradation during the initial healing phase is crucial for vascular repair and endothelialization.^29^ Further research is needed to evaluate long-term degradation after the coating has broken down.

Morphological observations showed that the PLLA layer remained continuous during immersion, maintaining a porous structure that limited crack initiation and propagation. Although time-resolved cross-sectional characterization was not conducted in this study, references suggest that similar coatings may appear denser after immersion. This apparent densification may be attributed to a combination of polymer chain relaxation, limited water absorption, and the deposition of inorganic species.^58^ Upon immersion, there is a partial rearrangement of the PLLA molecular chains, along with a reduction in free volume, which can lead to the contraction of pore structures. Additionally, the formation of degradation products, such as magnesium hydroxide and calcium phosphate compounds, may partially fill the surface pores, further contributing to the apparent densification of the coating.^59,60^ Furthermore, the corrosion behavior of PLLA-coated magnesium substrates is strongly influenced by the coating’s structure, including its porosity, thickness, and crystallinity.^61,62^ As a result, PLLA-coated ZK60 exhibited significantly reduced corrosion rates and suppressed formation of degradation products. Elemental analysis further demonstrated minimal deposition of phosphate species and an absence of calcium-containing products on PLLA-coated surfaces, even after prolonged immersion. This suppression of calcium accumulation is particularly relevant for cardiovascular stents, as calcium deposition is closely associated with vascular calcification and late-stage device failure.^63,64^

The observed controlled degradation behavior directly correlated with improved biological outcomes. Samples coated with PLLA showed sustained viability of endothelial cells. This effect is likely due to moderate magnesium ion release, minimized hydrogen evolution, and the stabilization of the local pH environment. These results align with previous research, which indicates that PLLA coatings effectively lower the corrosion rate of magnesium alloys by acting as a physical barrier and stabilizing the interface between the substrate and the physiological environment.^57,65^ Additionally, polymer coatings like PLLA have been shown to enhance cytocompatibility by reducing rapid ion release and preventing local alkalization during the degradation process.^29,66,67^ In addition, direct cell–material interaction studies showed that PLLA coating promoted stable endothelial adhesion, preserved cell morphology, and enhanced cell migration compared with uncoated ZK60.^68,69^ Accelerated gap closure on PLLA-coated substrates further indicates improved endothelial motility, which is critical for rapid re-endothelialization following stent implantation.^70^

The results of the current investigation support findings from previous studies. Samples coated with PLLA showed a significantly lower corrosion rate and an improved response from endothelial cells compared to uncoated samples. Overall, these findings demonstrate that PLLA coating acts not merely as a passive physical barrier, but as an effective regulator of the degradation–cell interaction interface. By simultaneously moderating corrosion kinetics and maintaining a biologically favorable microenvironment, PLLA surface modification represents a more effective and clinically relevant strategy than postextrusion annealing for improving the performance of biodegradable ZK60 magnesium alloys in cardiovascular stent applications.

## 5 Conclusion

This study systematically compared the effects of postextrusion annealing and PLLA surface modification on the mechanical behavior, degradation characteristics, and endothelial responses of ZK60 magnesium alloy. Postextrusion annealing altered intrinsic mechanical properties but showed limited capability in controlling degradation or improving endothelial performance under physiological conditions.

In contrast, PLLA coating effectively stabilized the degradation interface, significantly reducing the corrosion current density to approximately 22 μA/cm^2^ and lowering the corrosion rate to around 0.51 mm/year. In comparison, uncoated samples exhibited a corrosion current density ranging from 151 to 223 μA/cm^2^ and a corrosion rate between 3.46 and 5.09 mm/year. This enhancement in corrosion resistance was accompanied by improved endothelial responses, including a cell viability increase to about 140% and better cell adhesion and migration behavior. These results demonstrate that surface engineering exerts a more dominant influence than bulk heat treatment in regulating degradation-driven functional performance of extruded ZK60.

Overall, the findings highlight interface-focused surface modification as a more effective strategy for optimizing biodegradable magnesium-based cardiovascular stents, providing clear guidance for future material design and translational development.
